# Effectiveness of High-Fidelity Simulation in Training Emergency Medicine Physicians in Point of Care Ultrasonography in Pakistan: A Quasi-Experimental Study

**DOI:** 10.7759/cureus.8659

**Published:** 2020-06-17

**Authors:** Kiran Azizi, Madiha Ismail, Umaira Aftab, Badar Afzal, Asad Mian

**Affiliations:** 1 Emergency Medicine, Aga Khan University, Karachi, PAK; 2 Pediatric Emergency Medicine, Aga Khan University, Karachi, PAK

**Keywords:** pocus, emergency medicine physician, high fidelity simulation, skill training, efast

## Abstract

Background

Point-of-care ultrasound (PoCUS) is frequently utilized in emergency medicine (EM), with an extended-focused assessment with sonography in trauma (e-FAST) being the most widely used PoCUS modality. This modality is not only time- and cost-efficient, but it is highly accurate in the diagnosis and management of surgical patients in the emergency department, as well as being highly predictive of patient outcomes. Targeted training is essential to ensure a learner's confidence in image acquisition, interpretation, and translation of knowledge to making clinical decisions. High-fidelity simulation offers a uniquely safe and "mistake-forgiving" environment to teach and train medical professionals. The present study evaluated the effectiveness of a high-fidelity simulator to train EM physicians in e-FAST at a tertiary care teaching hospital in a lower-middle-income country.

Methods

This quasi-experimental study was performed at a state-of-the-art simulation center of a multidisciplinary university hospital in Karachi, Pakistan. Subjects were included if they were EM physicians who volunteered to participate and were available for the entire training and testing period. The educational intervention included lectures and hands-on practice on a high-fidelity simulator (SonoSim, Santa Monica, CA).

Knowledge and image interpretation on e-FAST were evaluated using a questionnaire, administered before and after the training course. Each participant's ability to acquire and interpret satisfactory images was assessed by experienced EM physicians and recorded. Participants were also administered a needs assessment survey and a course evaluation. Data were analyzed using IBM SPSS Statistics for Windows, Version 20.0 (Armonk, NY: IBM Corp.). All the tests were two-sided, and p-values ≤0.05 were considered significant. Baseline characteristics and outcome variables were recorded and compared by Wilcoxon signed-rank tests.

Results

A total of 31 EM physicians, 12 (38.7%) men and 19 (61.3%) women, were enrolled in the study, with 24 (77.3%) having one to three years of EM experience. Mean and percentage group performance improved from 6 and 40% before the intervention to 14.5 and 96.6% after the intervention (Z=4.867, p≤0.05). Most improvement in image acquisition on high-fidelity simulation was observed in the upper right quadrant of the suprapubic window (29/31; 93.5%), followed by the upper left quadrant (27/31; 87%) and the subxiphoid window (21/31; 67%). All 31 participants reported improvements in comfort and confidence level with PoCUS after attending the workshop.

Conclusions

EM physicians who attended a brief workshop incorporating simulation demonstrated improvements in knowledge and image acquisition skills in all domains tested. High-fidelity simulation training is an effective modality for training EM physicians in e-FAST.

## Introduction

Point-of-care ultrasound (PoCUS) is defined as ultrasonography at the patient's bedside performed in real time by a care provider [1]. Focused assessment with sonography for trauma (FAST) is an integrated, goal-directed, bedside examination performed to detect fluid, which is likely to be hemorrhage in cases of trauma [2]. In addition to pericardial and peritoneal windows, extended FAST (e-FAST) also includes an examination of the chest. Studies from the developed world have shown that e-FAST is an effective and sensitive technique for the detection of blunt abdominal trauma and that it is equally accurate when administered by radiologists and non-radiologists [3].

Similar to other aspects of physical examinations, PoCUS is dependent on the operator [4]. Confidence and competence in both image acquisition and interpretation are essential, as is knowledge of how to incorporate findings into clinical decisions [5]. Because optimal training of physicians is necessary, many institutions worldwide include PoCUS in their undergraduate and postgraduate training and curricula [6]. Little is known, however, about the ability of physicians to acquire e-FAST skills using high-fidelity simulators to diagnose life-threatening conditions in trauma patients [7,8]. Moreover, the feasibility and effectiveness of this program in a lower-middle-income country (LMIC) like Pakistan have not been determined [2,3]. The present study assessed the knowledge and skills of emergency medicine (EM) physicians after a brief training workshop on e-FAST, including high-fidelity simulators, in a tertiary care teaching hospital in Karachi, Pakistan.

## Materials and methods

This quasi-experimental study enrolled EM physicians, including postgraduate medical trainees (residents) and non-trainees (medical officers and senior medical officers), working in an urban tertiary care university hospital. The study was approved by the ethical review committee of Aga Khan University and was conducted in a simulation center located in a multidisciplinary university hospital in Karachi, Pakistan. All EM physicians at the hospital were invited to participate, with all those agreeing and enrolled providing written informed consent.

**Figure 1:**
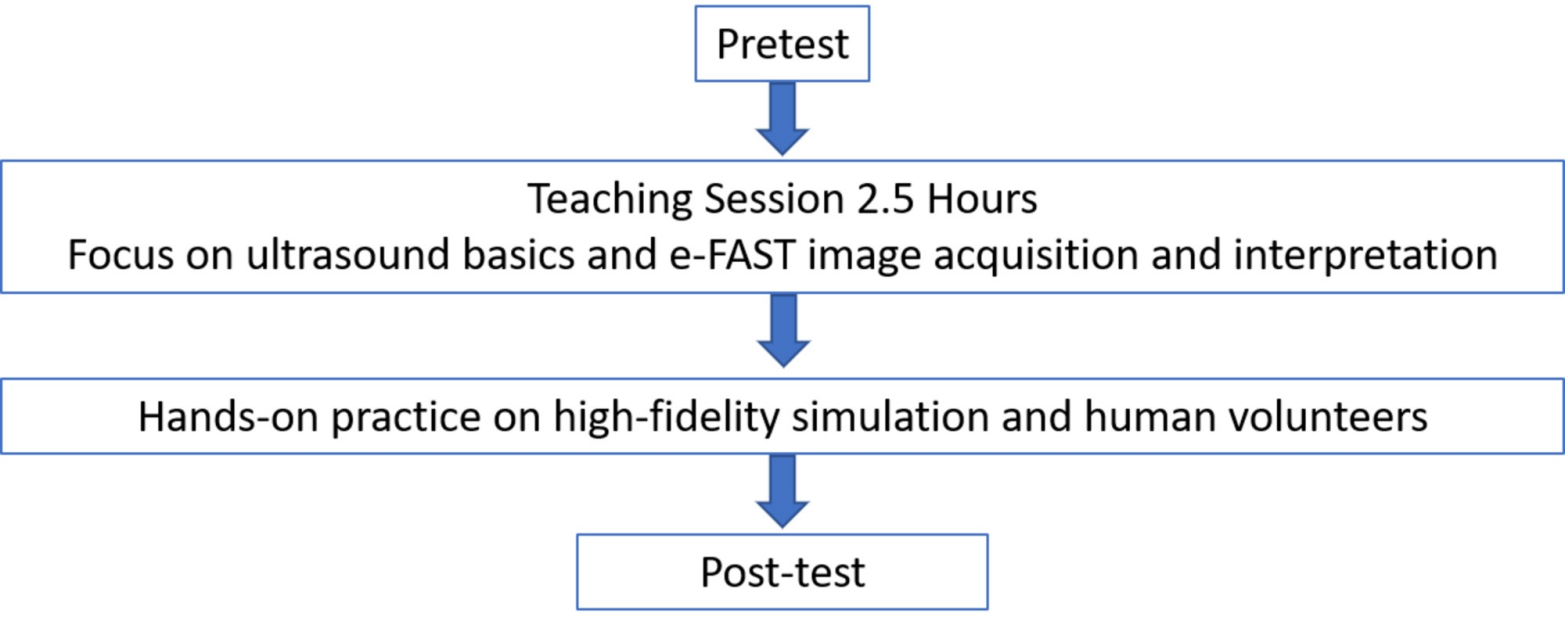
Flow diagram of the study e-FAST, extended-focused assessment with sonography in trauma.

Participants were eligible if they were EM physicians who volunteered to participate, and were available for the entire training and testing period. Each physician participated in a half-day (3.5-hour) workshop on PoCUS-eFAST, which included didactic lectures on knobology, knowledge of image acquisition, and knowledge of image interpretation, followed by a hands-on practice session and then assessment on a high-fidelity simulator (SonoSim, Santa Monica, CA) and by a human volunteer (Figure 1).

Knowledge regarding image acquisition and interpretation skills was evaluated by a questionnaire and by experienced EM physicians. The preintervention questionnaire included questions about demographic characteristics, need assessments, cognitive skills and interpretation of e-FAST images, and barriers to PoCUS practice. After the training session, the participants were administered the same questionnaire, which included questions about subjects' knowledge and feedback on the workshop. To assess changes in knowledge, each trainee's results before and after the training session were compared. Data were compiled anonymously by a blinded reviewer and analyzed statistically using IBM SPSS Statistics for Windows, Version 20.0 (Armonk, NY: IBM Corp.).

All statistical analyses were two-sided, with p-values ≤0.05 considered statistically significant. Normally distributed continuous variables were reported as mean ± standard deviation, and skewed variables were reported as median and range. Results obtained before and after the training session were compared by the Wilcoxon signed-rank test. Generalized estimation equation analysis was used to determine factors influencing the variability in scores before and after the intervention.

## Results

The study enrolled 31 qualified medical practitioners, including 12 (38.7%) men and 19 (61.3%) women, with 24 (77.4%) having one to three years of work experience. Eight (27.6%) were residents, 11 (38%) were medical officers, and 10 (34.5%) were senior medical officers. Only eight (26%) participants had previously received formal ultrasound training. Although 29 (93.6%) reported that an ultrasound machine was easily available in the emergency department, only three (9.86%) were using it routinely for FAST examinations. A total of 24 participants (77.4%) identified a lack of training, and 18 (58.1%) identified a lack of knowledge as the most important barriers to PoCUS utilization. Following training, all 31 (100%) self-reported improvement in levels of comfort and confidence with PoCUS, with 20 (64%) stating that ultrasound teaching sessions should take place on monthly (Table 1).

**Table 1:** Baseline characteristics of the study participants (N=31)

Variables	n (%)
Gender	
Male	12 (38.7)
Female	19 (61.3)
Physician level in the emergency department	
Resident	8 (27.7)
Medical officer	11 (37.9)
Senior medical officer	10 (34.5)
Duration practicing in the emergency department	
1–3 years	24 (77.4)
4-5 years	3 (9.7)
≥5 years	4 (12.9)

A comparison of scores before and after training showed significant improvements (Table 2), with mean group performance improving from 6 to 14.5, and percentage improving from 40% to 96.6% (Z=4.867, p<0.05 by Wilcoxon signed-rank tests; Table 3).

**Table 2:** Comparison of correct responses by participants to questions before and after the training session (N=31) e-FAST, extended-focused assessment with sonography in trauma; FAST, focussed assessment with sonography in trauma.

Questions	Before training session	After training session	P-value	Confidence interval
What does FAST stand for?	20 (64.5)	29 (93.5)	0.002	-6.01, -1.36
Name all four windows used for evaluation of free fluid in FAST exam	18 (58.1)	31 (100.0)	<0.001	4.81, 8.71
What does e-FAST stand for?	12 (38.7)	29 (93.5)	<0.001	4.58, 8.25
Which windows are added in e-FAST exam?	9 (29.0)	30 (96.8)	<0.001	5.31, 8.86
Ideal probe for FAST exam?	19 (61.3)	31 (100.0)	0.010	0.75, 5.67
Amount of blood detected by FAST	24 (77.4)	31 (100.0)	<0.001	3.47, 8.73
Most dependent point of peritoneal cavity	18 (58.1)	28 (90.3)	<0.001	1.98, 5.80
Pneumothorax sign on sonography	7 (22.6)	24 (77.4)	0.112	-0.41, 3.98
Identification of suprapubic window	12 (38.7)	30 (96.8)	<0.001	4.83, 8.18
Free fluid (suprapubic window)	18 (58.1)	30 (96.8)	<0.001	4.33, 8.01
Identification of Morrison’s pouch	14 (45.2)	30 (96.8)	<0.001	5.49, 8.52
Free fluid (Morrison’s pouch)	15 (48.4)	29 (93.5)	<0.001	4.01, 7.03

 

**Table 3:** Comparison of correct responses by participants to questions before and after the training session (N=31) * Wilcoxon signed-rank test. † Based on positive ranks.

Variables	Mean	Median	Standard deviation	Difference	Z-Score	P-value
Before training	6.58	6	4.02	251	4.867^†^	<0.001*
After training	14.67	15	0.65			

Generalized estimation equation analysis of factors influencing the variability in scores before and after intervention showed that high scores were more likely to be achieved by participants in their mid-level career stage, with work experience of four to five years (Table 4). Also, those identifying lack of training as the most significant barrier to PoCUS utilizations scored higher on postintervention tests (Table 4).

**Table 4:** Statistical comparison of test scores before and after the training session using a generalized estimating equation test model * Significant P-values. PoCUS, point-of-care ultrasound. S.E. β, standard error β.

Variables	β-coefficient (S.E (β))	P-value	Confidence interval
Duration practicing in the emergency department			
1–3 years	-	-	-
4–5 years	2.66 (0.88)	0.003*	0.92, 4.39
≥5 years	- .41 (0.94)	0.134	-3.27, 0.43
Physician level in the emergency department			
Resident	-	-	-
Medical officer	-2.20 (0.70)	0.002*	-3.57, -0.82
Senior medical officer	-0.66 (0.77)	0.389	-2.18, 0.85
In my opinion, “Lack of training” is the biggest barrier to utilizing PoCUS in the emergency department	2.08 (0.68)	0.002*	0.74, 3.42

 Physicians were inquired about the needs assessment of PoCUS in emergency department (Table 5).

**Table 5:** Needs assessment of PoCUS in the ED PoCUS, point-of-care ultrasound; FAST, focused assessment with sonography in trauma; IVC, inferior vena cava; CVP, central venous pressure; DVT, deep vein thrombosis; OB/GYN, obstetrics/gynecology; ED, emergency department.

PoCUS utilization in the ED	n (%)
An ultrasound machine is easily available in my department	29 (93.5)
I frequently use PoCUS	
Strongly disagree	3 (9.78)
Disagree	4(12.9)
Neutral	22(71.0)
Agree	2 (6.5)
Strongly agree	
I have received formal training on PoCUS in the past	
Yes	8 (25.8)
No	23 (74.2)
I frequently use ultrasound for FAST exam	
Strongly disagree	6 (19.4)
Disagree	8 (25.8)
Neutral	13 (41.9)
Agree	2 (6.5)
Strongly agree	1 (3.2)
Missing	1 (3.2)
I frequently use ultrasound to look for pneumothorax	
Strongly disagree	13 (41.9)
Disagree	7 (22.6)
Neutral	8 (25.8)
Agree	2 (6.5)
Strongly agree	-
Missing	1 (3.2)
I frequently use ultrasound to assess IVC collapsibility (fluid assessment)	
Strongly disagree	3 (9.7)
Disagree	4 (12.9)
Neutral	12 (38.7)
Agree	7 (22.6)
Strongly agree	4 (12.9)
Missing	1 (3.2)
I frequently use ultrasound for CVP insertion	
Strongly disagree	3 (9.7)
Disagree	2 (6.5)
Neutral	5 (16.1)
Agree	7 (22.6)
Strongly agree	13 (41.9)
Missing	1 (3.2)
I frequently use ultrasound to look for pulmonary edema (B lines)	
Strongly disagree	7 (22.6)
Disagree	4 (12.9)
Neutral	9 (29.0)
Agree	6 (19.4)
Strongly agree	4 (12.9)
Missing	1 (3.2)
I frequently use ultrasound for DVT	
Strongly disagree	15 (48.4)
Disagree	12 (38.7)
Neutral	3 (9.7)
Agree	-
Strongly agree	-
Missing	1 (3.2)
I frequently use ultrasound for OB/GYN	
Strongly disagree	18 (58.1)
Disagree	12 (38.7)
Neutral	-
Agree	-
Strongly agree	-
Missing	1 (3.2)
In my opinion, “shortage of time” is the biggest barrier to utilizing PoCUS in the ED	
Agree	19 (61.3)
Disagree	12 (38.7)
In my opinion, “lack of training” is the biggest barrier to utilizing PoCUS in the ED	
Agree	24 (77.4)
Disagree	7 (22.6)
In my opinion, “shortage of staff” is the biggest barrier to utilizing PoCUS in the ED	
Agree	6 (19.4)
Disagree	25 (80.6)
In my opinion, “limited knowledge by physicians” is the biggest barrier to utilizing PoCUS in the ED	
Agree	18 (58.1)
Disagree	13 (41.9)
This workshop has increased my level of comfort and confidence in PoCUS	
Strongly disagree	-
Disagree	-
Neutral	-
Agree	4 (12.9)
Strongly agree	27 (87.1)
How frequently should ultrasound sessions be held?	
Monthly	20 (64.5)
Quarterly	7 (22.6)
Half yearly	3 (9.7)
Yearly	1 (3.2)

**Figure 2:**
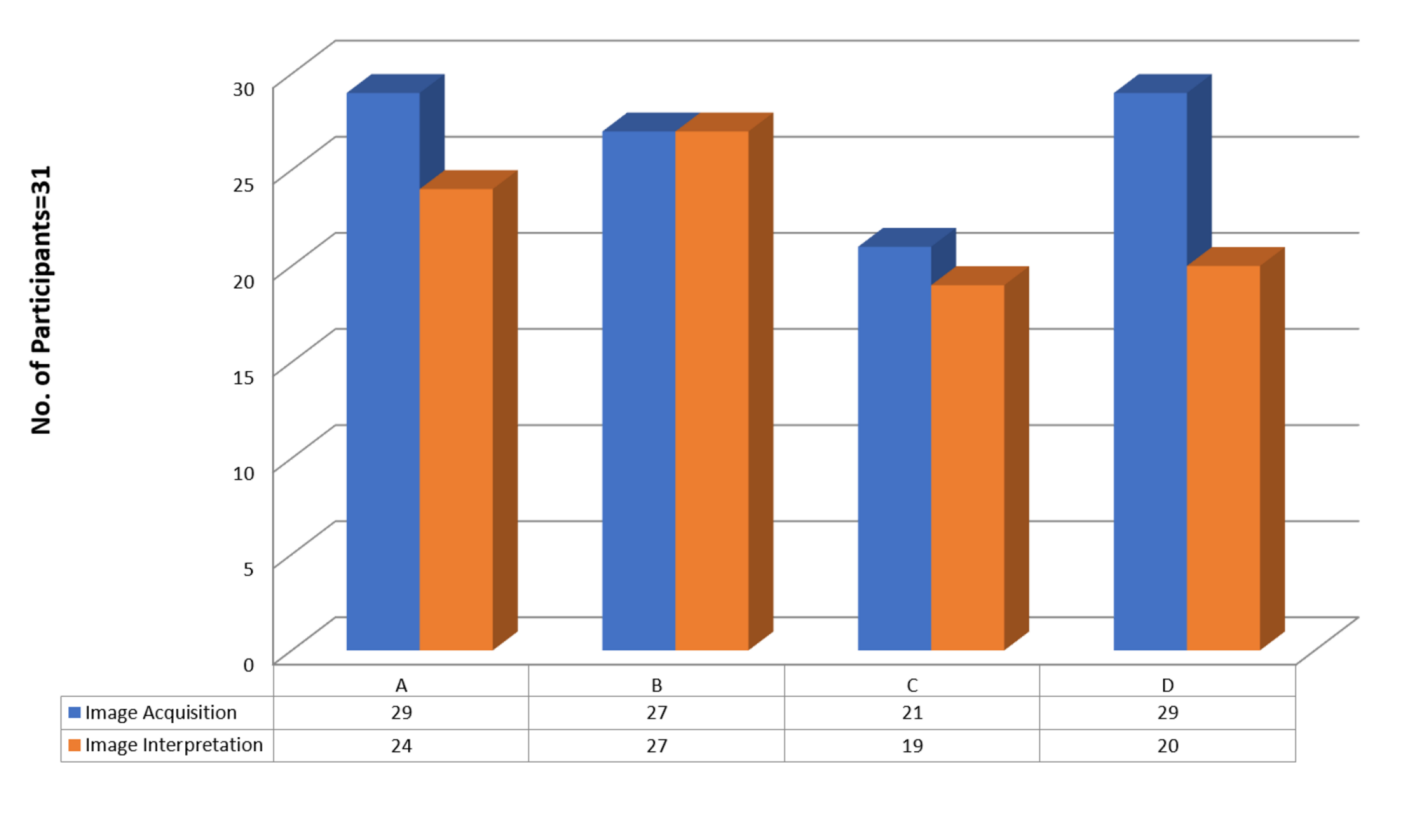
Correct image acquisition and interpretation, as determined by high-fidelity simulation

Participants were tested on four trauma scenarios using a high-fidelity simulator (SonoSim). Images of the right upper quadrant, left upper quadrant, and subxiphoid and suprapubic windows were accurately acquired by 29 (93.5%), 27 (87%), 27 (87%), and 29 (93.5%) participants, respectively, whereas images of these windows were correctly interpreted by 24 (77.4%), 27 (87%), 19 (61.3%), and 20 (64.5%) subjects, respectively (Figure 2).

**Figure 3:**
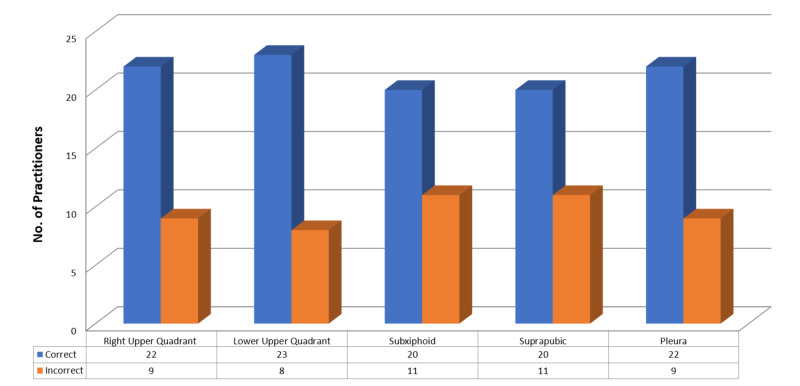
Correct image acquisition, as determined by an experienced emergency medicine physician

When evaluated by an experienced EM physician, images of the right upper quadrant, left upper quadrant, subxiphoid, suprapubic and pleural windows were accurately acquired by 22 (70.9%), 23 (74.2%), 20 (64.5%), 20 (64.5%), and 22 (70.9%) participants, respectively (Figure 3).

## Discussion

This study showed that a high-fidelity simulation-based workshop improved the knowledge and skills of emergency physicians performing e-FAST. Learners displayed a significant improvement in written post-training scores that assessed knowledge and image interpretation abilities. Although subjects participated in a half-day workshop focused on e-FAST, their post-training test scores were comparable to those of a three-day PoCUS workshop [9]. This is important for educators when planning skill-based simulation courses, as even a short course improved knowledge and interpretation of images by EM physicians. Secondary generalized estimation equation analysis showed that physicians with four to five years of experience did better on test scores than those with more than five years of experience, confirming earlier findings [9].

All 31 participants reported subjective improvements in comfort and confidence level regarding the use of eFAST at the bedside, comparable to previous results [10]. A positive correlation has been observed between confidence and knowledge [11]. Moreover, both can decay after initial training and can be regained after refresher courses [12].

Pre-training needs assessment found that, despite the availability of an ultrasound machine, it was mostly used for central venous line access and fluid assessment only, whereas other modalities, such as bedside echo and FAST, were mostly underutilized. This observation is not only consistent with other studies from LMICs but also highlighted the urgent need of integrating PoCUS into the EM residency curriculum in developing countries [8].

Similar to previous studies, the major barriers to PoCUS utilization identified by the participants in our were lack of training and limited knowledge [13,14]. The introduction of longitudinal teaching programs for both trainees and non-trainee physicians in EM may not only enhance their knowledge but result in better patient-centered outcomes [11,15].

Additional studies are needed to ascertain the optimal duration of high-fidelity-based simulation courses on PoCUS. In most developed countries, physicians participate in two- to three-day, multiple level sign-out courses in ultrasound [11]. However, two-hour sessions may be beneficial, with flattening of operators' learning curves after 10 to 30 examinations [16-18]. Moreover, written tests of knowledge regarding the use of PoCUS do not correlate with procedural skills, suggesting that evaluation of both skillsets may be necessary to tailor training [19].

Limitations

This study had several limitations. First, it was performed at a single academic center with a small group of participants, with all participants being invited volunteers. Secondly, we did not evaluate hands-on skills before the training session; therefore, improvements in image acquisition could not be evaluated. Also, retention and application of knowledge in clinical settings were not evaluated (Kirkpatrick's level 3 evaluation), and the confidence level was assessed only after the training session [20]. Although we observed a positive outcome, we did not compare outcomes after our 3.5-hour session with those after other courses of different lengths. Thus, an optimal course length requires further evaluation. Additional studies are needed to determine long-term retention of knowledge and skills after training and to determine whether knowledge gained in a simulation setting is used in real-life clinical practice.

## Conclusions

PoCUS is not being used to its full potential in emergency departments of LMICs. The major barriers are the lack of training and knowledge. High-fidelity simulation training is effective in training EM physicians in e-FAST, with a half-day workshop that included simulations resulting in significant improvements in written test scores that assessed cognitive and image interpretation abilities. Further studies are needed to determine whether knowledge gained in simulation settings can be translated into clinical practice.
